# The Wabanaki Approach to Treating Opiate Use Disorder among Indigenous North Americans

**DOI:** 10.1192/j.eurpsy.2022.1156

**Published:** 2022-09-01

**Authors:** L. Mehl-Madrona, B. Mainguy

**Affiliations:** 1 University of Maine, Native Studies/intermedia, Orono, United States of America; 2 Coyote Institute, Education, Ottawa, Canada

**Keywords:** Indigenous people, cultural healing, opiate use disorder, harm reduction

## Abstract

**Introduction:**

Addictions are prominent among indigenous people in North America in relation to historical and contemporary trauma.

**Objectives:**

We describe the approach emerging in our services for the five indigenous tribes of Maine (the Wabanaki Confederacy) for culturally sensitive treatment of opiate use disorder.

**Methods:**

In our auto-ethnographic approach, we introduce or re-introduce participants to cultural beliefs, values, and methods for treating addictions, inclusive of narrative methods (storytelling) which receive greater acceptance by indigenous and marginalized peoples. Indigenous philosophy states that we see the world using the stories that we have absorbed or constructed to explain our perceptions. Using substances is a story that is connected to poverty and adverse childhood events. We create new stories to develop a sense of agency, the sense that one’s actions can make a difference in one’s life.

**Results:**

We present the lessons learned and the results of our using this approach with a tribal population in Maine. Some key concepts include (1) reframing the person’s self-story about being addicted within a threat-power-meaning network, (2) working with stories about the spirit of the addiction and the consequences of ingesting spirit-laden substances without knowing their songs and protocols, (3) constructing future-self-narratives that explore right relationships and meaningful conduct, (4) constructing stories about the intergenerational transmission of addictions and exploring the question of “whom will be the recipient of your addiction?”

**Conclusions:**

We come to understand that the client sets their goals and defines what recovery means for them, which is the heart of a harm reduction approach.

**Disclosure:**

No significant relationships.

